# Pyroptosis-Mediated Damage Mechanism by Deoxynivalenol in Porcine Small Intestinal Epithelial Cells

**DOI:** 10.3390/toxins15040300

**Published:** 2023-04-19

**Authors:** Tae Hong Kang, Sangsu Shin, JeongWoong Park, Bo Ram Lee, Sang In Lee

**Affiliations:** 1 Department of Animal Science and Biotechnology, Kyungpook National University, Sangju 37224, Republic of Korea; 2 Research Center for Horse Industry, Kyungpook National University, Sangju 37224, Republic of Korea; 3 Department of Animal Biotechnology, Kyungpook National University, Sangju 37224, Republic of Korea; 4 Animal Biotechnology Division, National Institute of Animal Science, Rural Development Administration, Wanju 55365, Republic of Korea

**Keywords:** deoxynivalenol, intestinal epithelial cell, porcine, pyroptosis

## Abstract

Deoxynivalenol (DON) is known as a vomitoxin, which frequently contaminates feedstuffs, such as corn, wheat, and barley. Intake of DON-contaminated feed has been known to cause undesirable effects, including diarrhea, emesis, reduced feed intake, nutrient malabsorption, weight loss, and delay in growth, in livestock. However, the molecular mechanism of DON-induced damage of the intestinal epithelium requires further investigation. Treatment with DON triggered ROS in IPEC-J2 cells and increased the mRNA and protein expression levels of thioredoxin interacting protein (TXNIP). To investigate the activation of the inflammasome, we confirmed the mRNA and protein expression levels of the NLR family pyrin domain containing 3 (NLRP3), apoptosis-associated speck-like protein containing a caspase recruitment domain (ASC), and caspase-1 (CASP-1). Moreover, we confirmed that caspase mediates the mature form of interleukin-18, and the cleaved form of Gasdermin D (GSDMD) was increased. Based on these results, our study suggests that DON can induce damage through oxidative stress and pyroptosis in the epithelial cells of the porcine small intestine via NLRP3 inflammasome.

## 1. Introduction

Deoxynivalenol (DON), one of the various mycotoxins, is produced by *Fusarium* species and infects feed ingredients, such as corn, barley, and wheat. The contamination of grains with DON is exacerbated when exposed to unsuitable temperatures and humidity conditions during growing [1,2]. Human and farm animals are exposed through the intake of DON-contaminated cereal and feedstuff [3]. When domestic animals ingest DON-contaminated feedstuff, various symptoms are induced, such as immunotoxicity, changes in neuroendocrinology, anorexia, malabsorption, emesis, and weight reduction, according to exposure doses [4]. Among the diversity of farm animals, pigs are especially sensitive to DON due to bioavailability and rapid absorption [3,5]. DON has been known to damage vital organs, including the small intestine, kidney, spleen, and liver, through inflammation, oxidative stress, apoptosis, cell cycle arrest in the G2/M cell cycle phase, and cell signaling deregulation [6,7,8]. DON induces the dysfunction of protein and nucleic acid biosynthesis by binding to ribosomes, resulting in negative effects on cells. The small intestine and liver are organs with high protein turnover; therefore, these are mainly targeted by the cytotoxicity of DON [9]. Although the harmful effects of DON are well known, the molecular mechanism of the damage to the small intestine remains unclear.

Intestinal epithelial cells are attached to each other by junction complexes, such as tight junctions, adherens junctions, and desmosomes, from apical to basolateral [10]. Tight junctions (TJs) are the most apical components between epithelial cells, which serve to maintain cell polarity and structure and establish a barrier to regulate the paracellular movement of molecules for intestinal homeostasis [11]. Intestinal epithelial cells play two crucial functions: they serve as a transporter of nutrients and fluid and as a barrier function between the internal and external environment. These form a monolayer sheet, which is a dynamic and selective barrier, and transport molecules in two ways: the transcellular pathway (either across the cells) or the paracellular pathway (between cells) [12,13]. Under normal conditions, the maintenance of the intestinal epithelial cells (IECs) is important for development, normal growth, and disease prevention [14]. Dysfunction of the intestinal barrier is related to diverse diseases, such as celiac disease, inflammatory bowel disease (IBD), and irritable bowel syndrome (IBS), and this dysfunction is also associated with other organ diseases, including type I diabetes, autism, and multiple sclerosis [15]. An intestinal barrier function defect is influenced by specific environmental factors, which include pathogen infections, bacterial infections, diseases, pesticides, food additives, medication exposure, and mycotoxins. Mycotoxin is a factor of intestinal barrier dysfunction via junctional complex damage, cell commitment, and reactive oxygen species (ROS) production [16,17].

Intracellular ROS consist of a class of highly reactive chemicals, including singlet oxygen (^1^O_2_), hydrogen peroxide (H_2_O_2_), superoxide (O_2_^−^), hypochlorite (CIO^−^), hydroxyl radical (·OH), and peroxynitrite (ONOO^−^) [18]. ROS play an important role in normal physiology and maintaining homeostasis in cells; however, excessive ROS can induce oxidative stress, which is defined as the disequilibrium of the pro-/antioxidant balance and causes mitochondrial damage [19,20,21]. According to various studies, oxidative stress is related to the pathophysiology of inflammation, cancer, fibrosis, apoptosis, and reperfusion injury [22]. Oxidative stress, through the accumulation of excessive ROS, is involved in damage to intestinal homeostasis, which results in the impairment of the antioxidant system, damaging the electron transport chain and energy metabolism enzymes, thereby leading to cell death [23]. Oxidative stress induced by ROS, including nitric oxide, hydroxyl radicals, and superoxide radicals, is known to cause IBD and apoptosis in the gastrointestinal tract [24,25,26]. Pyroptosis, one of the diverse types of cell death, is an inflammatory form of programmed cell death by inflammasome activation [27]. Pyroptosis occurs in various organs, including the intestine or liver, and releases inflammatory cytokines, such as IL-18 and IL-1β. The released inflammatory cytokines contribute to inflammation and disease progression [28,29]. Inflammasomes are multimolecular protein complexes consisting of apoptosis-associated speck-like proteins containing a caspase recruitment domain (ASC), nucleotide-binding oligomerization domain (NOD)-like receptors (NLRs), and CASP1, and they act as an important factor in the inflammatory response to endogenous damage and exogenous pathogens, such as cytosolic double-stranded DNA, bacterial infection, crystals, and toxins [30]. Inflammasomes facilitate the activation of caspase 1 (CASP1), leading to the activation of Gasdermin D (GSDMD), thereby triggering cell membrane pore formation, which induces pyroptosis [31,32,33].

This study aimed to determine whether DON induces ROS and pyroptosis and confirm the molecular mechanism of pyroptosis in porcine small intestinal epithelial cells.

## 2. Results

### 2.1. DON Induced ROS Production in Porcine Intestinal Epithelial Cells

**Figure 1:**
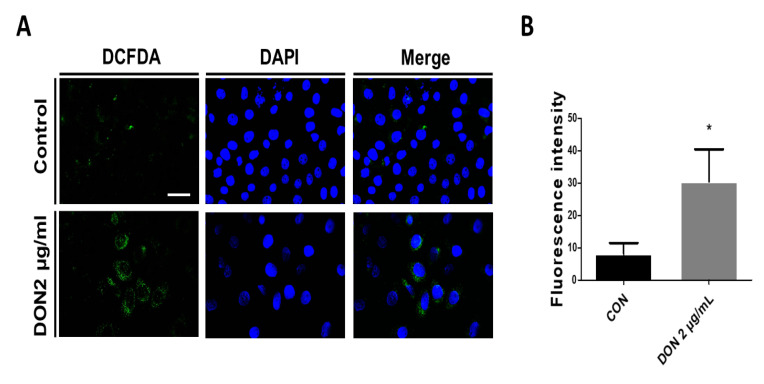
Deoxynivalenol (DON) induces reactive oxygen species in porcine intestinal epithelial cells. (**A**) IPEC-J2 cells were stained with 10 µM of 2, 7-dichlorodihydrofluorescein (DCFDA). (**B**) Quantity analysis of intensity of the DCFDA. Fluorescence intensity was measured using Image J software 1.44. Error bars indicate the mean ± standard deviation (SD) of the analysis performed in triplicate. * *p* < 0.05. Nuclei were stained with 4′,6-diamidino-2-phenylindole (DAPI; in blue). The image was obtained by fluorescence microscopy. Scale bar = 40 µm.

In our previous study, the 50% inhibitory concentration (IC50) in IPEC-J2 cell viability was shown at a DON concentration of 2 μg/mL. Therefore, a concentration of DON 2 μg/mL was used in further study. To determine whether DON induces the production of ROS, we performed cell staining using 2,7-dichlorofluorescein. After IPEC-J2 cells were incubated for 24 h with 2 μg/mL of DON, the ROS were evaluated in comparison with the control group (Figure 1A,B). These data show that DON induced the accumulation of ROS in the small intestine epithelial cells.

### 2.2. DON Decreased the Expression of Antioxidant-Related Genes in Porcine Intestinal Epithelial Cells

**Figure 2:**
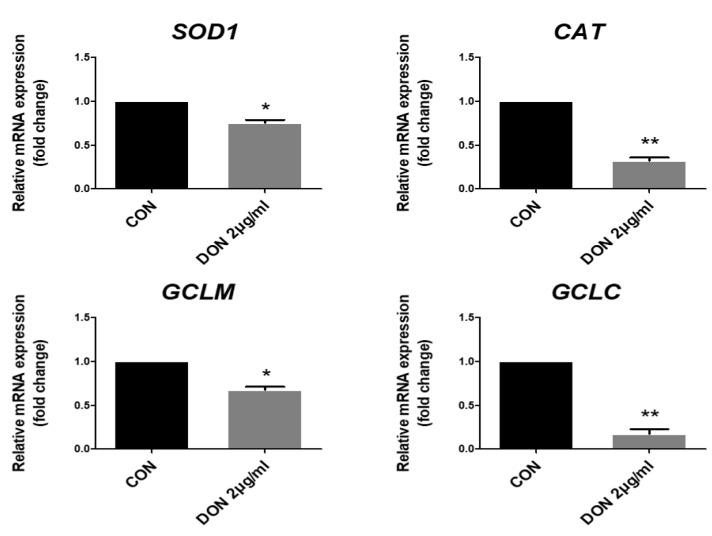
Deoxynivalenol (DON) decreased the expression levels of antioxidant genes. The mRNA levels of antioxidant-related genes (CAT, SOD1, GCLC, and GCLM) treated with 2 µg/mL DON compared with untreated control (CON) cells. Error bars indicate the mean ± standard deviation (SD) of the analysis performed in triplicate. * *p* < 0.05, ** *p* < 0.01.

To investigate the collapse of the pro-/antioxidant balance through the DON-mediated accumulation of ROS, we confirmed the expression of antioxidant-related genes, such as superoxide dismutase 1 (SOD1), catalase (CAT), Glutamate-cysteine ligase modifier subunit (GCLM), and Glutamate-cysteine ligase catalytic subunit 9 (GCLC). When IPEC-J2 cells were treated with DON, antioxidant-related genes were markedly decreased. These data indicate that DON induced the collapse of the pro-/antioxidant balance through the excessive accumulation of ROS (Figure 2).

### 2.3. Effect of Thioredoxin Interacting Protein, Inflammasome, and Pro-Inflammatory Cytokines in the mRNA Levels after DON Treatment

**Figure 3:**
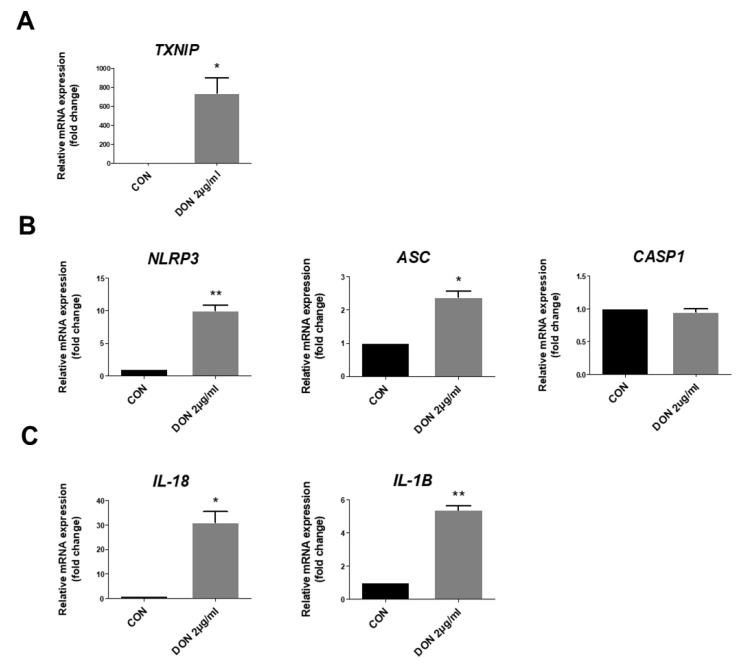
Deoxynivalenol (DON) induced the activation of TXNIP, inflammasomes, and pro-inflammatory cytokines. (**A**) The relative quantitative expression of TXNIP of DON-treated cells compared to control (CON) cells. (**B**) The mRNA expression levels of inflammasomes (NLRP3, ASC, and CASP1) of DON-treated cells compared with the control group. (**C**) The mRNA expression levels of pro-inflammatory cytokines (IL-1β and IL-18) of DON-treated cells compared with the control group. Data are expressed as mean ± standard deviation (SD) of analysis performed in triplicate. * *p* < 0.05, ** *p* < 0.01.

We affirmed the mRNA expression levels of TXNIP and validated the mRNA expression levels of inflammasomes, which were consistent with NLRP3, ASC, and CASP1. We confirmed the mRNA expression levels of pro-inflammatory cytokines such as interleukin-18 (IL-18) and interleukin-1β (IL-1β). Expression levels of TXNIP when treated with 2 μg/mL DON were remarkably increased compared with the untreated group (Figure 3A). Additionally, DON treatment significantly increased the mRNA expression levels of NLRP3 and ASC compared with the control group. However, the mRNA expression level of CASP1 showed no difference (Figure 3B). The mRNA expression levels of the pro-inflammatory cytokines, including IL-18 and IL-1β, were significantly increased in the IPEC-J2 cells treated with DON (Figure 3C).

### 2.4. Effect of TXNIP, Inflammasome, and Pro-Inflammatory Cytokines in the Protein Levels after DON Treatment

**Figure 4:**
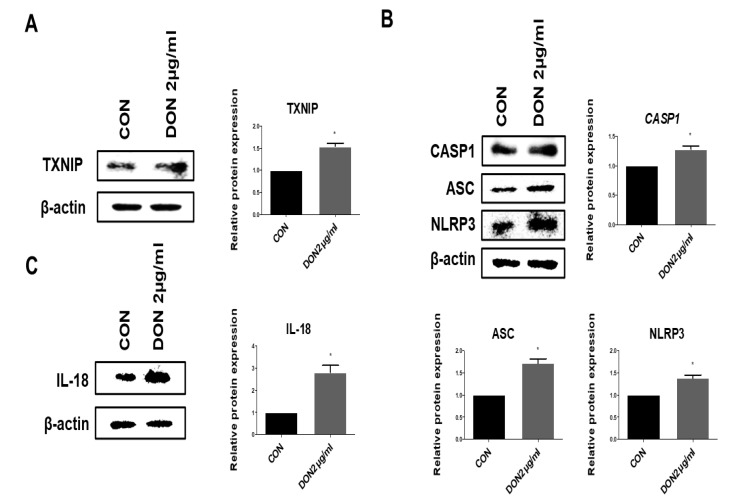
Deoxynivalenol (DON) treatment affected the protein expression levels of TXNIP, inflammasomes, and pro-inflammatory cytokine. (**A**) Protein expression level of TXNIP was evaluated by Western blotting assay. (**B**) Protein expression levels of inflammasomes (NLRP3, ASC, and CASP1) were evaluated using Western blotting assay. (**C**) Protein expression level of IL-18 was evaluated by Western blotting assay. The protein levels were normalized by β-actin. Data are expressed as mean ± standard deviation (SD) of analysis performed in triplicate; a representative example of the Western blotting image is shown. * *p* < 0.05.

We verified the activation of TXNIP, inflammasomes, and pro-inflammatory cytokines in the protein levels after treatment with DON. After DON treatment, the relative protein expression level of TXNIP was remarkably higher than that of the untreated cells (Figure 4A). The inflammasome complex, including NLRP3, ASC, and CASP1, were also significantly increased in the IPEC-J2 cells treated with DON (Figure 4B). IL-18 pro-inflammatory cytokines were also significantly increased in the IPEC-J2 cells treated with DON compared to the untreated cells (Figure 4C). These data reveal that the activation of the inflammasome complex, including ASC, CASP1, and NLRP3, increased inflammatory cytokine expression after treatment with DON.

### 2.5. DON Induced Pyroptotic Cell Death in IPEC-J2 Cells

**Figure 5:**
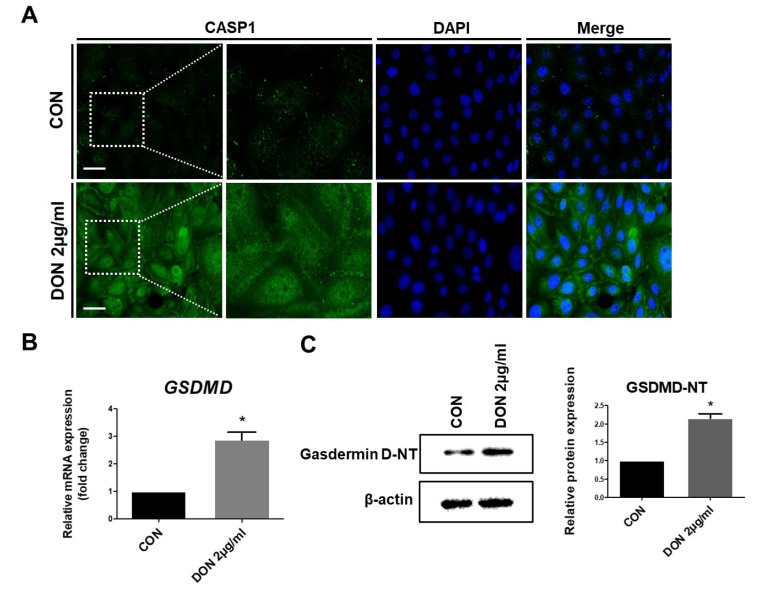
Activation of CASP1 and GSDMD induces pyroptotic cell death when treated with deoxynivalenol (DON). (**A**) IPEC-J2 cells were stained for CASP1 using immunocytochemistry. Nuclei were stained with 4′, 6-diamidino-2-phenylindole (DAPI; in blue). The images were obtained by using fluorescence microscopy. Scale bar = 40 µm. (**B**) Relative quantitative expression of GSDMD compared to the control group. (**C**) Protein expression level of GSDMD-NT was detected by using Western blotting. Data are expressed as mean ± standard deviation (SD) of analysis performed in triplicate; a representative example of the Western blotting image is shown. * *p* < 0.05.

To verify pyroptosis cell death in IPEC-J2 cells when treated with DON, we performed immunocytochemistry, quantitative real-time polymerase chain reaction (PCR), and Western blotting. When treated with DON, CASP1 expression was detected compared to the control group (Figure 5A). The relative mRNA expression level of GSDMD was significantly higher than that of the untreated cells (Figure 5B). Likewise, the relative protein expression level of GSDMD was notably increased compared with the untreated cells (Figure 5C). Based on these data, it is evident that DON induces pyroptotic cell death in the porcine small intestinal epithelial cells.

## 3. Discussion

DON, one of the various mycotoxins, is prevalent in food and feed [34]. It occurs in grains, such as wheat, barley, and corn, during harvesting and is not easily eliminated, leading to ongoing contamination [35]. DON is of major interest because of its high incidence at the global scale and high occurrence in the feedstuff in livestock diets [36]. It also remains in the body of livestock, resulting in its detection in livestock products such as meat, milk, and eggs [37]. DON has been especially known to be harmful to pigs; the reason for this is the high bioavailability, glucuronidation metabolism for excretion, lack of detoxification gut microorganisms, and long clearance time [38,39,40]. After the ingestion of DON, it may be rapidly absorbed and accumulate in the small intestinal tract, leading to antifeeding, vomiting, necrosis of the digestive tract, and a decrease in reproductive performance [41,42]. DON exposure causes diverse effects in various body systems, such as the gastrointestinal, neuroendocrine, and reproductive systems, intestinal toxicity, and results in growth impairment and immune dysfunction [36,43]. The intestinal epithelium, undergoing continuous cell renewal, is a major target for DON, which binds to the peptidyl transferase of active ribosomes, leading to the inhibition of protein translation. This exposure can trigger IBDs, gut disorders, and diarrhea [44]. Previous studies have suggested that DON can induce apoptosis, inflammation, oxidative stress, disruption of intestinal barrier function, DNA damage, cell cycle arrest, and deregulation of cell signaling [45]. To investigate DON-mediated damage in IPEC-J2, we studied the mechanism of small intestine damage by DON-induced pyroptosis.

To investigate whether DON induces the production of ROS and oxidative stress, we performed DCFDA staining and qPCR. The present study indicates that DON induced the production of ROS and oxidative stress in porcine small intestine epithelial cells. Diverse biochemical processes and physiology oxidation lead to the production of ROS, which are unstable molecules, including singlet oxygen, superoxide, hydroxyl radicals, and hydrogen peroxide [46]. Generally, these play a major role in intensifying the defense against pathogens or serve as signaling molecules [45]. However, the prolonged accumulation of ROS causes non-specific damage to lipids, proteins, and nucleic acids and induces oxidative stress, which is the disproportion in the pro-/antioxidant balance [47,48]. ROS including O_2_^·−^. hydroxyl radical (HO·), and the oxidant H_2_O_2_ are produced from the general oxidative metabolism of oxygen in the cell, and processes that induce the separation of electron transport can increase the production of ROS in the mitochondria. In addition, endoplasmic-reticulum-bound enzymes, the external plasma membrane, and cytoplasmic enzyme systems can also induce the production of ROS [48]. High oxygen metabolism levels increase ROS production and can enhance the permeability of the mitochondrial membrane, resulting in the release of ROS into the cytosol [47]. Previous reports have indicated that the production of ROS by DON causes injuries to the small intestine, such as inflammation and apoptosis [49]. Consistent with these previous results, our study also indicates that DON causes the production of ROS in the small intestinal epithelial cells. Antioxidants contribute to the regulation of ROS levels through the control of ROS-producing enzymes and free-radical-eliminating mechanisms, thereby preventing free radical damage by offering electrons to damaged cells [50,51]. Antioxidants can be classified as enzymatic and non-enzymatic antioxidants. Enzymatic antioxidants operate to eliminate ROS. In a multistep process, the presence of cofactors, including manganese, iron, copper, and zinc, antioxidants, can change an oxidative product to hydrogen peroxide and then to water. Non-enzymatic antioxidants, including vitamin E, vitamin C, carotenoids, and glutathione, operate to disturb free radical chain reactions [52]. SOD is the main antioxidant enzyme that detoxifies superoxide into hydrogen and oxygen by converting it to water and oxygen [22,53]. In addition, SOD1 especially initiates the cytoprotective pathway involving gene transcription from toxic stimulation. When ROS such as O_2_^·−^ -generating compounds, and H_2_O_2_ are accumulated in the cell, SOD1 is translocated from the cytosol into the nucleus and regulates the transcription of ROS-induced stress, homeostasis of cellular redox state, and cellular-stress-related genes [53]. CAT exists in almost all organisms and is a normal antioxidant enzyme that uses either iron or manganese as a cofactor and catalyzes the degradation of or decline in hydrogen peroxide [54]. CAT has a protective function that prevents oxidative damage in the cell by decomposing hydrogen peroxide to oxygen and water [55]. Glutamate-cysteine ligase composed of GCLM and GCLC acts as a rate-limiting enzyme for the de novo synthesis of glutathione, which protects against oxidative stress [56,57]. In this study, ROS levels were increased (Figure 1), and antioxidant-related gene (SOD1, CAT, GCLM, and GCLC) expression levels were decreased by treatment with DON (Figure 2). Previous reports have shown that DON causes ROS production and oxidative stress in various cells [18,58]. We suggest that DON induces oxidative damage in the small intestine epithelial cells through the excessive accumulation of ROS.

**Figure 6:**
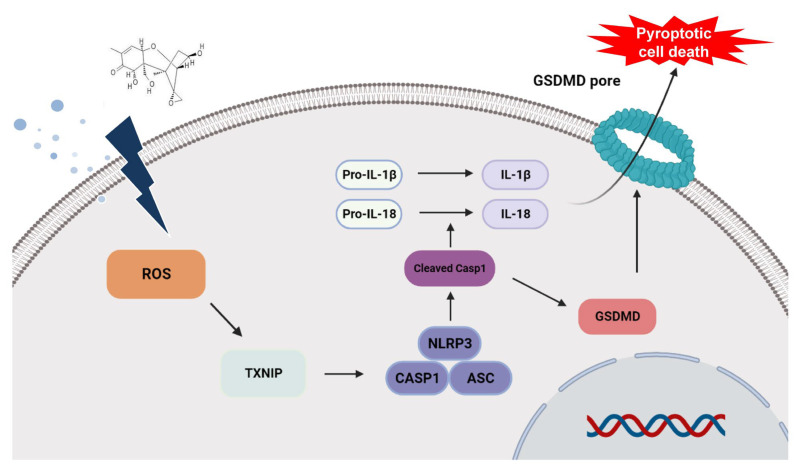
Schematic illustration of the current mechanism of deoxynivalenol (DON)-mediated pyroptosis via reactive oxygen species (ROS)-dependent NLRP3 inflammasome in the small intestine epithelial cells. DON induces the accumulation of ROS and the activation of thioredoxin interacting protein (TXNIP), inflammasome complex, and Gasdermin D (GSDMD), resulting in pyroptosis.

Pyroptosis, a lytic programmed cell death, induces excessive inflammatory damage through the activation of the inflammasome complex consisting of NLRP3, ASC, and CASP1 [59]. TXNIP is an endogenous negative regulator of TRX, which is a redox-active protein and has a defense function against ROS through involvement in reactive oxidative metabolism in the normal condition. TXNIP is separated from the TXNIP-TRX complex when ROS accumulation by pathogen-associated molecular patterns (PAMPs) and damage-associated molecular patterns (DAMPs) leads to cell damage. The separated TXNIP interacts by binding with the NLRP3 inflammasome and leads to GSDMD-mediated pyroptotic cell death [60]. In this study, we investigated the expression of DON-induced ROS-dependent TXNIP and confirmed the activation of the inflammasome complex, including NLRP3, ASC, and CASP1, in the porcine intestinal epithelial cells (Figure 3 and Figure 4). The NLRP3 inflammasome is important in the immune defense system against fungal, viral, and bacterial infections and has been implicated in immune disorders such as autoimmune diseases, IBD, diabetes, myocardial infarction, and atherosclerosis [61,62]. In our study, the CASP1 mRNA and protein expression patterns differed. This may be due to the distinction between the mRNA and protein turnover rates or post-transcriptional regulation. DON-mediated CASP1 expression potentially prefers to exist as an activated protein than in a post-transcription regulatory state. Once the inflammasome complex is activated, NLRP3 operates as a sensor molecule and induces the structural change in ASC through homotypic PYD-PYD interactions, resulting in the activation from pro-caspase-1 to CASP1 [63]. It also induces the mature form of cytokines, such as IL-18 and IL-1β, from pro-IL-18 and pro-IL-1β and cleaves the Gasdermin-N amino-terminal and Gasdermin-C carboxy-terminal domains of GSDMD. GSDMD-Nterm induces the formation of a membrane pore, and then, the cells are swelled and undergo osmotic pressure changes, which collapse the cell membrane, resulting in the release of inflammatory cytokines, including IL-18 and IL-1β, and then, inflammatory cytokines gather more inflammatory factors, leading to inflammatory injury [64,65,66,67]. To verify whether DON causes pyroptotic cell death, we examined the CASP1 expression of the NLRP3 inflammasome complex by DON and confirmed the expression of GSDMD-Nterm (Figure 5). Previous reports indicated that GSDMD-Nterm serves to regulate pyroptosis by the cell membrane pore, and then, inflammatory cytokines are released through the membrane pore [68]. Likewise, our study also showed that the expression of GSDMD-Nterm and inflammatory cytokines was increased. In summary, DON induces ROS accumulation, and then, activated TXNIP activates the NLRP3 inflammasome. Cleaved CASP1 induces activated form GSDMD and inflammatory cytokines, cleaved form GSDMD-Nterm causes the cell membrane pore, and then inflammatory cytokines are released. Finally, this molecular mechanism results in pyroptotic cell death (Figure 6). Based on these results, we suggest that DON can induce oxidative stress, and it causes pyroptosis via NLRP3 inflammasome-induced activation of GSDMD in porcine small intestinal epithelial cells. This study may improve the understanding of the mechanism of small intestine damage through the intake of DON-contaminated feedstuff.

## 4. Conclusions

In conclusion, DON induces the production of ROS and causes oxidative stress. Antioxidant-related genes are also reduced by DON. TXNIP is activated by DON-mediated excessive ROS, which activates CASP1 by activating NLRP3 inflammation. This induces mature forms of cytokines, including IL-18, from pro-IL-18 and cuts the Gasdermin-N amino and Gasdermin-C carboxy-terminal domains of GSDMD. GSDMD-Nterm forms pores in the cell membrane. As a result, inflammatory cytokines are released through the pore, causing inflammatory forms of programmed cell death. This study improves the understanding of DON toxicity in the small intestinal epithelial cells, and the results of this study may be beneficial in mitigating DON-induced damage by targeting the NLRP3 inflammasome pathway, which will be helpful in research to improve pig productivity.

## 5. Materials and Methods

### 5.1. Cell Culture and Treatment

The IPEC-J2 cell line was obtained from DMSZ. IPEC-J2 cells were cultured using Dulbecco’s Modified Eagle Medium (DMEM) (Cat. NO. 11965118, Thermo Fisher Scientific, Wilmington, DE, USA) with 10% fetal bovine serum (FBS) (Cat. No. 12483020, Thermo Fisher Scientific, Wilmington, DE, USA) and 1% penicillin–streptomycin at 37 °C in a CO_2_ incubator. Following the cell viability results of our previous study, DON-treated concentration used was 2 µg/mL [69].

### 5.2. Cell Passage and Cryopreservation

IPEC-J2 cells were seeded 2 × 10^5^ in 6-well plates and were incubated for 3 days. IPEC-J2 cells were washed using a phosphate-buffered saline (PBS) (Cat. No. 10010049, Thermo Fisher Scientific, Wilmington, DE, USA). After 0.25% trypsin-DETA (Cat. No. 25200056, Thermo Fisher Scientific, Wilmington, DE, USA) was added to cells for 5 min at 37 °C in a CO_2_ incubator, the medium was supplemented and centrifuged for 5 min at 1200 rpm. Following that, IPEC-J2 cells were resuspended in medium and seeded in 6-well plates. 

The 0.25% trypsin-EDTA was supplemented in IPEC-J2 for 5 min at 37 °C in a CO_2_ incubator and added to the medium at a ratio of 1:1. Collected cells were centrifuged for 5 min at 1200 rpm. The Cell banker (Cat. No. 11889, Amsbio, Seoul, Republic of Korea) was added to the collected pellet and resuspended using a pipette. The cells were transferred in cryogenic vials and stored at −80 °C deep freeze. 

### 5.3. Intracellular ROS Detection

IPEC-J2 cells were seeded at density of 1 × 10^4^ in a 24-well plate and cultured for 32 h. Cells were then incubated overnight in DMEM and cultured with 2 µg/mL DON for 24 h. Cells were washed with PBS and fixed in 4% paraformaldehyde (PFA) for 15 min. After cells were fixed, they were treated with 10 µM 2,7-dichlorodihydrofluoroscein diacetate (DCFDA) (Cat. No. C400, Thermo Fisher Scientific, Wilmington, DE, USA) for 30 min in a 37 °C incubator. Experiments were performed in triplicate. Stained images were obtained by fluorescence microscope (Korealabtech, Gyeonggi-Do, Republic of Korea). Fluorescence intensity was quantitated using Image J.

### 5.4. Quantitative Real-Time PCR and Western Blotting

After IPEC-J2 cells were seeded at density of 2 × 10^5^ in 6-well plates and cultured for 32 h, they were added with a non-FBS medium and incubated overnight. IPEC-J2 cells were treated with 2 µg/mL DON for 24 h. To extract the total RNA, an AccuPreP Universal RNA extraction kit (Cat. No. K-3141, BioNEER) was used. The cDNA was synthesized from 1 μg of the total RNA using a DiaStar Kit. To design the primers for the target genes for qPCR, the Primer 3 program was used (http://frodo.wi.mit.edu). qPCR was performed as per the following protocol: 95 °C for 3 min, followed by 40 cycles of 15 s at 95 °C, 56–58 °C for 15 s, and 72 °C for 15 s. qRT-PCR data were normalized to the expression of glyceraldehyde 3-phosphate dehydrogenase (GAPDH). The fold changes in mRNA expression levels were calculated using the 2^−ΔΔCt^ method [70]. Experiments were performed in triplicate. The primer sequence of genes was shown in Table S1.

After IPEC-J2 cells were seeded at density of 2 × 10^5^ in 6-well plates and cultured for 32 h, they were replaced with a non-FBS medium and incubated overnight. IPEC-J2 cells were treated with DON concentration of 2 µg/mL for 24 h. Proteins were extracted using a lysis buffer (Cat. No. #9803, Cell signaling Technology, Danvers, MA, USA) supplemented with a protease inhibitor cocktail (Cat. No. P3100-001, GenDEPOT, Katy, TX, USA). Concentration of the protein was determined using a Pierce BCA Protein Assay kit (Cat. No. 23225, Thermo Fisher Scientific, Wilmington, DE, USA). After electrophoresis for 1 h on a 9% polyacrylamide gel, the protein samples were transferred to a membrane for 90 min at 100 V. After blocking for 1 h, the membrane was treated overnight with the corresponding primary antibody, including anti-TXNIP (Polyclonal; 1:1000; Cat. No. LS-B5829, LSBio, Seattle, WA, USA), anti-NLRP3 (Polyclonal; 1:1000; Cat. No. 19771-1-AP, Proteintech, Chicago, IL, USA), anti-ASC (Polyclonal; 1:2000; Cat. No. 10500-1-AP, Proteintech, Chicago, IL, USA), anti-CASP1 (Polyclonal; 1:1000; Cat. No. 22915-1-AP, Proteintech, Chicago, IL, USA), anti-IL-18 (Monoclonal; 1:1000; Cat. No. BMS1042, Thermo Fisher Scientific, Wilmington, DE, USA), and anti-GSDMD-N (1;1000; Cat. No. DF12275, Affinity, OH, USA), anti-beta actin (Cat. No. ab8227, Cambridge, UK). The membrane was treated with the secondary antibody for 1 h and visualized using an enhanced chemiluminescence reagent. The protein band images were obtained by using the ChemiDoc imaging system. 

### 5.5. Immunofluorescence Staining of Porcine Intestinal Epithelial Cells

IPEC-J2 cells were seeded at a density of 1 × 10^4^ on gelatin-treated coverslips of a 24-well plate. After the cells were washed with PBS, 4% paraformaldehyde was used to fix the cells for 15 min at room temperature. Following that, the cells were blocked for 1 h and treated overnight with the corresponding primary antibody at a ratio of 1:200. After washing with PBS three times for 5 min, they were incubated with goat anti-Rabbit (488; Cat. No. ab150077, Thermo Fisher Scientific, Wilmington, DE, USA) at a ratio of 1:500 in the dark for 1 h. Following that, DAPI (Cat. No. H-1200, Vector Laboratories, Burlingame, CA, USA) was dropped onto the coverslips, and it was mounted for imaging. The images were obtained with a fluorescence microscope (Korealabtech, Gyeonggi-Do, Republic of Korea).

### 5.6. Statistics

To determine the significant differences between the control and treatment groups, all data were analyzed with a *t*-test in SAS software 9.4. The mean ± standard error (SE), indicated by the error bars, of the analysis performed in triplicate was provided. Significant differences were indicated by the following symbols: * *p* < 0.05, ** *p* < 0.01, and were estimated using the Duncan multiple range test.

## Data Availability

Not applicable.

## Supplementary Materials

The following supporting information can be downloaded at: https://www.mdpi.com/article/10.3390/toxins15040300/s1, Table S1: List of primer.
