# Supplementary material for: Pyroptosis-Mediated Damage Mechanism by Deoxynivalenol in Porcine Small Intestinal Epithelial Cells
Source: Toxins (Basel). 2023 Apr 19;15(4):300. doi: 10.3390/toxins15040300 (PMC10146237; doi:10.3390/toxins15040300)
Supplement: Supplementary file 1 [file toxins-15-00300-s001.zip › toxins-2265683-supplementary.pdf]

toxins

MDPI

Supplementary Materials: Pyroptosis-Mediated Damage Mechanism by Deoxynivalenol in Porcine Small Intestinal Epithelial Cells

Tae Hong Kang, Sangsu Shin, JeongWoong Park, Bo Ram Lee and Sang In Lee

Table S1. List of primer.

Gene symbol	Description	Accession No.		Sequence (5'-3')	Sequence size	annealing temperature
SOD1	Superoxide dismutase 1	NM_001190422	Forward<br>Reverse	GAG ACC TGG GCA ATG TGA C<br>GAG GGA ATG TTT ACT GGG TGA	228	56°C
GCLM	Glutamate-cysteine ligase modifier subunit	XM_001926378	Forward<br>Reverse	CTT GCC TCT TGC TGT GTG AT<br>CGA TGT CAG GGA TGC TTT C	145	56°C
GCLC	Glutamate-cystein ligase catalystic subunit	XM_021098556	Forward<br>Reverse	CGT CTT TAC TGG GGG AAA AC<br>TTT ATC GCT TCG TCT GGA AA	148	56°C
CAT	Catalase	NM_214301	Forward<br>Reverse	GGC TTT TGG CTA CTT TGA GG<br>AGG GTC ACG AAC TGT GTC AG	148	56°C
IL-18	Interleukin18	NM_213997	Forward<br>Reverse	GCA TCA GCT TTG TGG AAA TG<br>GAA AAG GAC TTG GTC GTT CAG	179	56°C
IL-1β	IL-1beta	NM_001305893	Forward<br>Reverse	GAA CAA GAG CAT CAG GCA GA<br>TGG CAT CAC AGA CAA AGT CA	176	58.2°C
NLRP3	NLR pyrin domain containing 3	NM_001256770	Forward<br>Reverse	TCC TGA GCA ACA ACC AGA AG<br>TGT GAG ACA GCA ACT GAC CA	143	56°C
TXNIP	Thioredoxin interacting protein	NM_001044614	Forward<br>Reverse	TTG GAG GAA AGA CAG GAA AGA<br>AAC AAA ACC CCG AAT CAA AG	223	56°C
GSDMD	Gasdermin D	XM_021090506	Forward<br>Reverse	CAG AAG GAG GTG GAG GTC AC<br>GTC CCA GTC AGA ACC AAT CAC	186	58.7°C
ASC	apoptosis-associated speck-like protein containing a card	XM_003124468	Forward<br>Reverse	AGC AGA CAA CAA ACC AGC AC<br>GAA GAG CCT CCT CAT TTT GG	175	57.5°C
CASP1	Caspase 1	NM_214162	Forward<br>Reverse	CTG GCA TTT GTG GGA AGA A<br>ATC CAT ACG ACC CCT TGC T	174	56°C
GAPDH	Glyceraldehyde-3-phosphate dehydrogenase	NM_001206359	Forward<br>Reverse	ACA CCG AGC ATC TCC TGA CT<br>GAC GAG GCA GGT CTC CCT AA	150	56°C
